# P-376. Impact of a preoperative in-person assessment (PIPA) on Hip and Knee Replacement Surgical Site Infections (SSI)

**DOI:** 10.1093/ofid/ofae631.577

**Published:** 2025-01-29

**Authors:** Anupama Neelakanta, lauren Fitts, Kristin Fischer, Jessica Layell, Shelley Kester, Vignesh Rajan, Catherine Passaretti

**Affiliations:** Atrium Health, Charlotte, North Carolina; Atrium Health, Charlotte, North Carolina; Atrium Health, Charlotte, North Carolina; Atrium Health, Charlotte, North Carolina; Atrium Health, Charlotte, North Carolina; Atrium Health, Charlotte, North Carolina; Advocate Health, Charlotte, NC

## Abstract

**Background:**

SSI results in increased mortality, morbidity, length of stay and healthcare costs. SSI prevention is complex and preoperative optimization is an essential component. We aim to assess the impact of a preoperative in-person assessment (PIPA) on odds of SSI.

**Figure:**
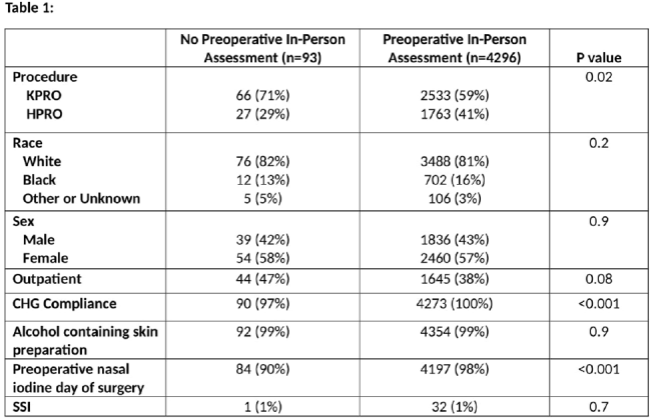

**Methods:**

We performed a retrospective study of 4389 elective hip (HPRO) and knee (KPRO) replacement surgeries performed between April 2022 through December 2023. Demographics, clinical risk factors, procedural data including SSI prevention bundle compliance, and data on completion of a PIPA were collated from the electronic health record and merged with infection preventionist (IP) SSI data obtained through routine surveillance by trained IPs using standard definitions. Patients with SSI present at the time of surgery were excluded. Patients who had a PIPA were instructed to bathe with chlorhexidine (CHG) for 5 days prior to surgery but there were no other differences in practice between the groups. Descriptive statistics were used to compare patients with and without a PIPA before surgery. We used logistic regression to investigate the relationship between PIPA and SSI adjusting for demographic and clinical factors.

**Figure:**
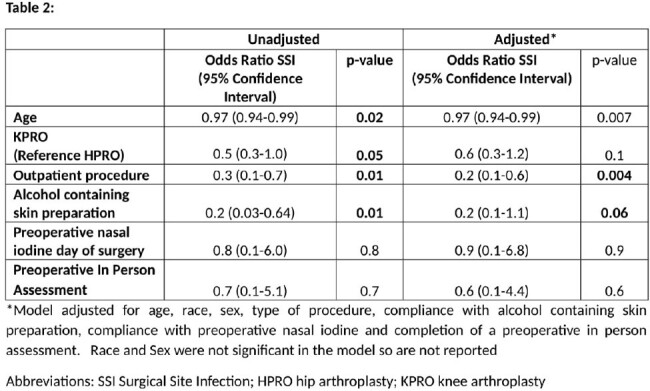

**Results:**

4296 (98%) of patients completed a PIPA. KPRO procedures and compliance with CHG bath and nasal iodine administration in the preoperative area were significantly higher in patients who had a PIPA. Rates of SSI were not significantly different between those who completed a PIPA and those who did not. (Table 1) In the univariate analysis, undergoing KPRO, having an outpatient procedure, increased age, and use of alcohol containing skin preparation were associated with lower odds of SSI (Table 2). After adjustment for age, outpatient status, alcohol containing skin prep, nasal care and PIPA, only age and outpatient procedure were associated with lower odds of SSI. Completion of a PIPA had no impact on SSI.

**Conclusion:**

PIPA did not impact odds of SSI however compliance with certain IP process measures such as nasal iodine and CHG was better in PIPA group. Alcohol containing skin prep is associated with improved SSI outcomes and should be encouraged. Limitations included single center study and small number of infections so more data needed to see impact of PIPA on SSI.

**Disclosures:**

**All Authors**: No reported disclosures

